# Burden of gastrointestinal cancers and problem of the incomplete information; how to make up the data? 

**Published:** 2016

**Authors:** Abdolhamid Sharifian, Mohamad Amin Pourhoseingholi, Ahmadreza Baghestani, Nastaran Hajizadeh, Sepideh Gholizadeh

**Affiliations:** 1 *Gandy General Hospital, Tehran, Iran *; 2 *Gastroenterology and Liver Diseases Research Center, Research Institute for Gastroenterology and Liver Diseases, Shahid Beheshti University of Medical Sciences, Tehran, Iran*; 3 *Department of Biostatistics, Shahid Beheshti University of Medical Sciences, Tehran, Iran*

**Keywords:** Gastrointestinal cancers, Burden, Mortality, Incidence, Registration

## Abstract

Cancer registration is an important source for measuring the burden of cancer in a population. In practice, however, quite frequently incorrect patients are registered or data items can be inaccurately recorded or not recorded at all. Also the process or quality of these registrations varies among countries. In this paper, we briefly discussed some statistical techniques including; Mortality and Incidence Analysis Model (MIAMOD), Prevalence and Incidence Analysis Model (PIAMOD), Bayesian Inference and Capture-recapture methods, which provide tools to re-correct the incomplete or misclassified cancer statistics with regards to gastrointestinal cancers.

## Introduction

 Cancer is one of the major leading causes of many disorders, death, and disabilities in the world (1). Among all cancers, gastrointestinal cancers (GI cancers) present an interesting pattern in distribution over the world. GI cancers are the leading health problem in the world and their burdens are increasing in many countries (2). There are two major projections to measure the burden of cancers including: mortality and incidence. These statistics are important to monitor the effects of screening programs, earlier diagnosis and other prognostic factors (3). Also, these statistics are useful to agencies, which are responsible for the provision of health and oncology services, continuing therapy, treatment of subsequent disabilities, medical consultations, etc. 

Cancer registration is an important source for measuring the burden of cancer in a population. Cancer registry is a systematic collection of any data regarding cancers, including: patient history, diagnosis, treatment, and status for every cancer patient. Population-based cancer registries monitor the frequency of new cancer cases every year in well-defined populations.

Data provided by these registries can be used to guide policy makers in order to set up cancer prevention programs. To be useful, data in a medical registry must be of good quality. However, in practice, quite frequently incorrect patients are registered or data items can be inaccurately recorded or not recorded at all (4). If any data on any variable from any participant is not present, the researcher is dealing with missing or incomplete data. 

**Figure 1:**
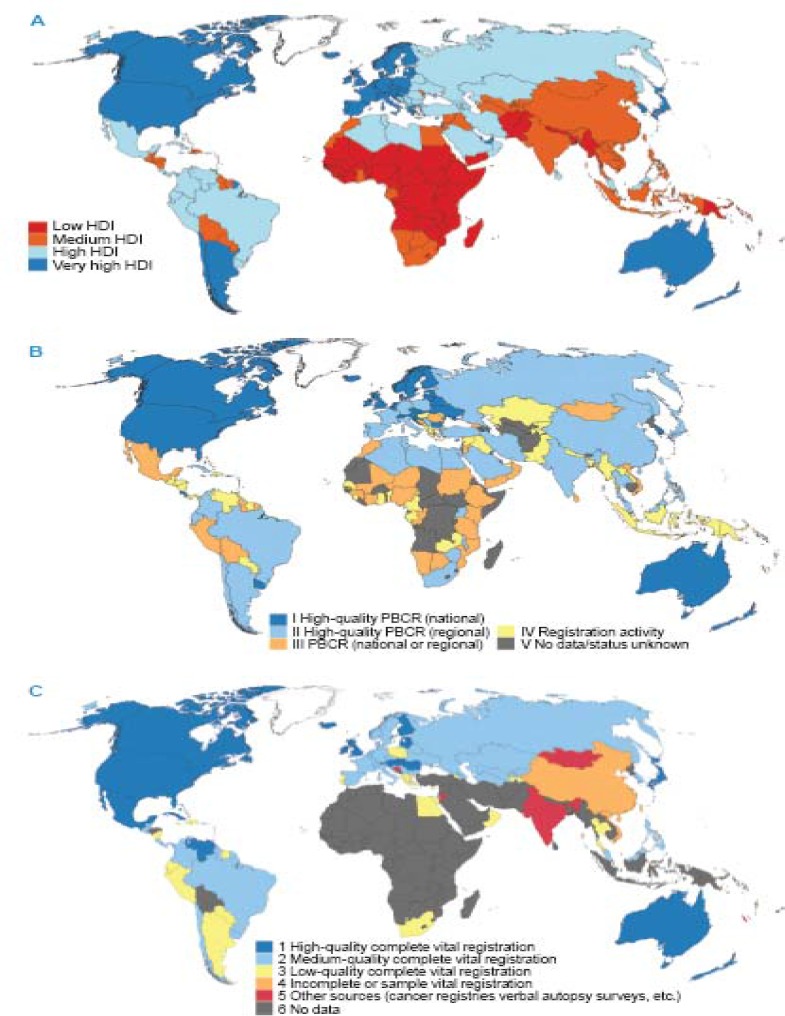
Global maps depicting (A) the development level of individual countries, according to the four-level Human Development Index (HDI), based on quartiles, for 2012; (B) status of population-based cancer registries (PBCRs), as of mid-2013; (C) status of vital registration systems, as of mid-2013

Many countries around the world have established cancer registries to record and collect data on cancer incidence (5). However, the process and quality of these registrations vary among countries. Figure 1 (6) compares the present levels of the national Human Development Index (HDI), versus available sources of cancer incidence and mortality data, which revealed that the quality of cancer registrations or access to the national registry systems are corresponding to the level of HDI. Countries with high or very high HDI, have almost developed their registration systems over the past decades to provide the information required in planning and evaluating cancer control plans (6). On the other hand, the situation is different for countries, with low HDI, mostly located in sub-Saharan, South Asia and some Latin-American countries; in these regions, both vital and cancer registration systems are not completed yet. The current low cancer incidence rates in these countries might be due to incomplete registration as well as incomplete diagnosis of cancer patients (7). The low incidence is not the only result of incomplete cancer registration. It may also be the case that those patients that are missed entirely are different in some way in terms of survival outcomes to those that are caught by the registration process (8). This is similar for mortality data. Mortality statistics can be achieved either via prospective active follow-up (which is almost expensive and potentially biased due to losses to follow-up), or via linkage to a regional or national death registry (9). But the incomplete enumeration of persons in a census and undocumented information maybe leads to incomplete mortality rates (10). A simulation study on GI and non-GI cancers showed that the incomplete death statistics also affect the population-based cancer survival. Also, when registry cover is not close to 100% completeness, long-term relative survival estimates and their comparison across populations must be interpreted with much caution (11). 

Some information on the cancer profile can be deduced from statistics that derived from other data sources, including: hospital discharge statistics or pathology department records. These systems have been developed in many Asian and Latin-American countries. However, the picture that emerges is often quite a biased one and much care is needed in the interpretation of the data. In hospital-based source, the information is incomplete due to patient attendance at a given hospital. For pathology-based source, data set is constructed from laboratory-based surveillance. Therefore, they could not reflect the complete image of total population (5). 

In the absence of registry system or in the case of incomplete misclassified data, some statistical models would serve as the flexible techniques to re-estimate the projections of the cancer burden. 

**MIAMOD/ PIAMOD Technique**

The Mortality and Incidence Analysis Model (MIAMOD) was developed to provide incidence, prevalence and mortality estimates, as well as projections, using mortality and patients’ survival information for national or regional levels (12). Since mortality data are available for the entire nation, and survival could be calculated from cohort studies or hospital documents. The MIAMOD method (as a back-calculation approach) can be used to calculate regional and national estimates of incidence and prevalence. This method is based on the relationships linking mortality and prevalence to incidence and survival, and has been widely applied to derive regional and national cancer burden estimates. The MIAMOD method receives as an input age specific mortality data for a specific calendar year (or a set of calendar years) and a specific cancer site of interest, age specific all causes mortality and population size (for the same years), as well as an estimate of a patient’s survival by age. Then, model results to expected incidence, mortality and prevalence, with projections to a chosen time period.

The other same technique, the Prevalence and Incidence Analysis Model (PIAMOD) estimates prevalence from incidence and survival by fitting a parametric incidence model to the incidence data (13). This method is useful to project prevalence in time. MIAMOD/PIAMOD methods have been used to estimate and project colorectal cancer (14, 15) and stomach cancer, at state level or for the rest of country’s population (16). 

**Bayesian Inference**

Bayesian inference is a method in statistics in which, prior information is used to update the probability of a hypothesis as evidence. Bayesian inference is closely related to subjective probability, often called Bayesian probability. Bayesian inference derives the posterior probability as the result of observed data and a prior probability. Bayesian modeling would be employed to estimate cancer incidence or mortality. Several studies calculated cancer incidence, using an age-period-cohort (APC) model with Bayesian approach (17). Also, a back-calculation based on a Bayesian approach was developed, that estimates the age-specific cancer incidence per year from age-specific cancer mortality (18, 19).

Besides, Bayesian modeling could be used in the case of misclassification. Generally, two approaches are recommended in statistical literature for misclassified statistics; the first is using a small validation sample (20) and the second is Bayesian analysis. In validation sample approach, one should cover a small sample of population and registers the validated information regarding the misclassified subject. For example, if the problem is to estimate the true mortality rate projection of gastric cancer, the researcher should prepare documented death records or certificates of a specific population using verbal autopsy in to find the misclassified rate and the results would be generalized to the rest of the population. In the second approach, subjective prior information (on at least some subset of the parameters) can be used to re-estimate misclassified statistic (21). By this method, one can correct the incidence or mortality underestimation due to the misclassified registry for the total of the population. For instance, Stamey et al. used Bayesian approach in data consisting of the number of deaths due to cancer and non-cancer, among residents of Hiroshima and Nagasaki, Japan, who experienced the atomic bomb disaster (22). Also, these models were employed to re-estimate the GI cancer mortality, including colorectal cancer and liver cancer (22, 23). The important assumption in this technique is to select true prior information regarding the misclassified parameter, which could make changes in the results that extended to the rest of the data. 

**Capture-recapture methods**

When several incomplete lists are available, using capture-recapture methods are recommended for reducing the costs of disease registration as well as reducing bias in incidence estimations. Capture-recapture methods have also been recommended for comparing population subgroups. Modeling the effect of intervening variables presents better estimations of population size, therefore solves many problems of the estimation of population size (24). Cancer registry completeness can be evaluated by independent case ascertainment, capture-recapture, or death-certificate methods (25).

The capture-recapture method is a sampling technique originally developed for ecological studies and then adapted to epidemiological studies, including confirmation the completeness of the data recorded in cancer registries (26, 27). The methodology is simple; a portion of the population is captured (from a list of registry, real population, etc.) and marked. In the next step, another portion is captured and the number of marked individuals from first sampling will be proportional to the number of marked individuals in the whole population to estimate the total population size. In brief, this method involves modeling the overlap between two or more lists of individuals from the target population, and using this model to predict how many additional individuals were unseen. To avoid bias in the estimate, the sources of data collection must be independent (28). 

This method was used to estimate the gastric cancer incidence in Tehran, revealed that the incidence estimated by capture-recapture method is about three times higher than the incidence reported by the registry sources (29). 

Also, there are other same methods, including Lincoln-Petersen method (which derived to estimate the number of cases that have not yet been observed, based on two independent sets of observed cases) and Chapman estimator (that could be applied for small sample size or small with less biased than Lincoln-Petersen estimation) with similar methodology (30). 

## Conclusion

In the past few decades, developing countries have had an increase in the burden of GI. In some countries, the vital registration data systems have covered only a part of the country and all deaths (or incidences) are not registered. Cancer registries are urgently needed in developing countries because the cancer burden is usually poorly known (31). In the absence of the reliable registration system, researchers and health policy makers could employ statistical models to revise the data. Theses statistical techniques would serve to makeup the gaps due to incomplete information and estimate the projections of burden. Today, software and packages are developed for these modeling. However, these models need true assumptions, which could be dismissed due to the nature of incomplete statistics.
